# Impact of Intraoperative Ergonomic Microbreaks on Surgeons' Well‐Being and Performance: A Systematic Review

**DOI:** 10.1002/wjs.70490

**Published:** 2026-08-01

**Authors:** Gabriel Heierli, Fabian Grass, Luís C. Pereira, Jean Pellegrini, David Fuks, Dieter Hahnloser, Martin Hübner

**Affiliations:** ^1^ Department of Visceral Surgery Lausanne University Hospital (CHUV) and University of Lausanne (UNIL) Lausanne Switzerland; ^2^ Department of Musculoskeletal Medicine Lausanne University Hospital (CHUV) and University of Lausanne (UNIL) Lausanne Switzerland; ^3^ Faculty of Biology and Medicine University of Lausanne (UNIL) Lausanne Switzerland

**Keywords:** ergonomics, microbreaks, physical and mental performance, surgeons, work‐related pain

## Abstract

**Background:**

Surgeons face a considerable risk of work‐related musculoskeletal disorders (WMSDs). Repetitive movements and poor ergonomic conditions can cause pain, particularly in the back, neck, arms, and shoulders. WMSDs not only impact surgeons' quality of life but may also affect surgical performance, absenteeism, and healthcare costs. To reduce WMSDs, intraoperative microbreaks (MBs) with active stretching exercises have been proposed. The aim of this review was to study ergonomic intraoperative MB interventions and their effect on surgeons' symptoms, well‐being and performance and to define the features of efficacious interventions.

**Method:**

A systematic review of clinical trials evaluating physical or psychological impact of intraoperative microbreaks on surgeons was conducted in MEDLINE, Embase and Google Scholar until October 2024. Outcomes of interest included pain, discomfort, fatigue, dexterity, mental focus, stress as well as objective measures.

**Results:**

Ten clinical trials (284 study subjects) were retained for final analysis (8 crossover trials and 2 randomized controlled trials). Methodology and assessed outcomes varied considerably between studies. Similarly, interventions were heterogenous in terms of duration, interval, or set of exercises. 6 out of the 10 trials showed at least one improved outcome after the implementation of MBs. The most frequently assessed outcomes were pain and discomfort (improved in 5/8 studies), mental focus (improved in 3/3 studies), and accuracy (improved in 1/3 studies). Five studies evaluated operative duration, all showing no prolonged operating time associated with MBs.

**Conclusion:**

Microbreaks might have a positive impact on surgeons' well‐being and performance. There is a clear need though for standardization of interventions and outcome measures. The long‐term effects of microbreaks and the most efficacious intervention need yet to be defined.

## Graphical abstract

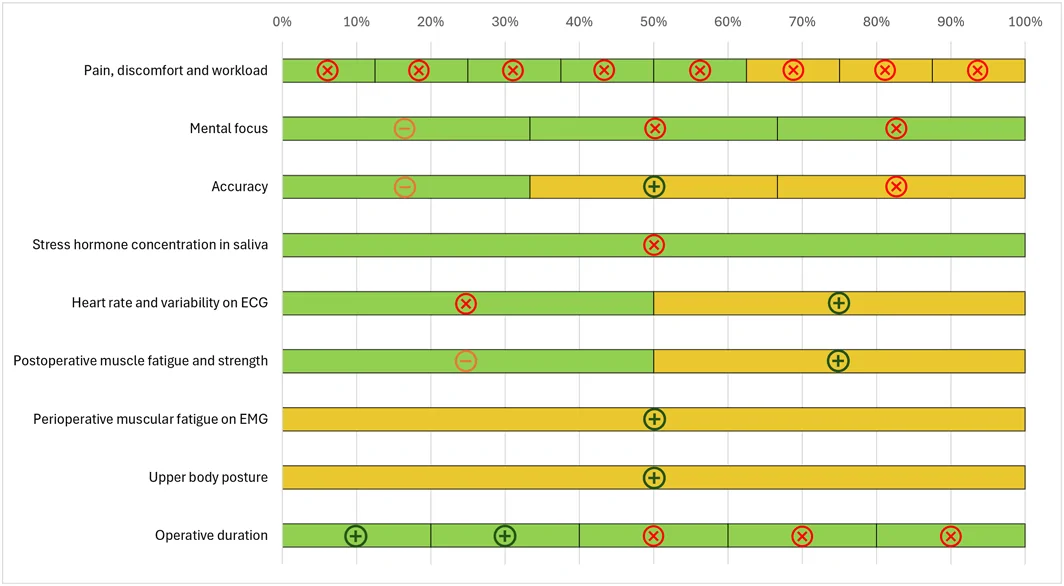

**Abbreviations**
MB(s): microbreak(s)
OR: operating room
WMSDs: work‐related musculoskeletal disorders

## 1 Introduction

The well‐known Latin adage “Medice, cura te ipsum” (“Physician, heal thyself”) has long served as a reminder to healthcare professionals to care for their own well‐being. Yet, surgeons report a high prevalence of work‐related musculoskeletal disorders (WMSDs) [1, 2, 3], even higher compared to high‐risk workers like labor‐intensive populations [4]. These WMSDs are associated with personal, circumstantial or occupational factors which are partially modifiable. Prolonged awkward positions, standing in ergonomically poor postures, repetitive movements, and poor surgical tool handle design [5] are important contributive factors. The most reported pain sites are the back, neck, arm and shoulders [2, 6]. Numerous studies have highlighted the magnitude of the problem [3, 6, 7].

These occupational injuries not only impact surgeons' quality of life but also patient care and healthcare costs. Several studies have shown that these injuries may result in higher absenteeism [3], reduced surgical volume [3, 8], earlier retirement [7, 9], and decreased self‐rated performance [8]. In addition to these WSMDs, surgeons' bad ergonomics may also lead to fatigue [10], loss of accuracy [11] and impaired mental focus [12].

Despite these issues being well documented, less than one‐third of surgeons seeks medical help for their symptoms [6] and less than 40% of surgeons report their musculoskeletal occupational injuries [8], to some extent related to “training in an environment that discourages complaints about stress and fatigue” [13].

Several studies focused on intraoperative microbreaks (MBs, short breaks during surgery without breaking the sterile field, ideally associated with active muscle stretching) to counter not only WMSDs, but also to improve other metrics like precision, fatigue, and performance. Some of these studies demonstrate encouraging results and have been included in clinical recommendations [14]. However, the suggested protocols are quite disparate and have been tested in various settings with diverse outcome measures.

The principal aim of this study was to provide a comprehensive literature overview regarding the impact of MBs on surgeons' well‐being and performance.

Secondary aims were to determine the “optimal microbreak” and to identify potential obstacles for MBs implementation in clinical routine.

## 2 Material and Methods

The methodology of this study followed the PRISMA recommendations [15] and a 24‐step guide [16] for systematic review and meta‐analysis. The review protocol was registered in the PROSPERO database (registration number: CRD420251148145).

The inclusion criteria were as follows: the target study population consisted of surgeons or surgical trainees, regardless of subspecialty. Eligible studies included clinical trials with a control group in which no MBs were performed. The interventions of interest were intraoperative MBs lasting ≤ 5 min, performed without breaking the sterile field. Only studies evaluating the actual performance of microbreaks, rather than merely providing information about them, were included. Eligible studies should report on physical, psychological, and performance‐related outcomes.

Exclusion criteria were systematic reviews and meta‐analyses, veterinary surgeries, ergonomic interventions other than MBs or mixed with MBs, no control group, and breaks lasting > 5 min.

A search equation using the PICO method [17] was created (Table 1).

**TABLE 1:** Search equation table following the PICO method (MEDLINE nomenclature is shown in this table as an illustration).

	Population	Intervention	Comparison	Outcomes
Concept	Surgeons	Microbreaks	No microbreaks	Pain Musculoskeletal pain Fatigue Surgical precision Dexterity Mental focus Stress Physical performance Discomfort
MeSH terms (PubMed)	“Surgeons”[Mesh] “Intraoperative Period”[Mesh]	“Muscle stretching Exercises”[Mesh] “Rest”[Mesh]	—	“Musculoskeletal diseases/prevention and control”[Mesh]
Free terms (PubMed)	“Surgeon*”[tiab]	“Breaks”[tiab] “Break”[tiab] “Microbreak*”[tiab] “Micro‐break*”[tiab] “Micropause*”[tiab] “Micro‐pause*”[tiab] “Ergonomic break*”[tiab] “Intraoperative break*”[tiab] “Intra‐operative break*”[tiab] “peroperative break*”[tiab]	—	“pain”[tiab] “fatigue”[tiab] “precision”[tiab] “dexterity”[tiab] “focus”[tiab] “stress*”[tiab] “Physical performance*”[tiab] “discomfort*”[tiab]

**FIGURE 1:**
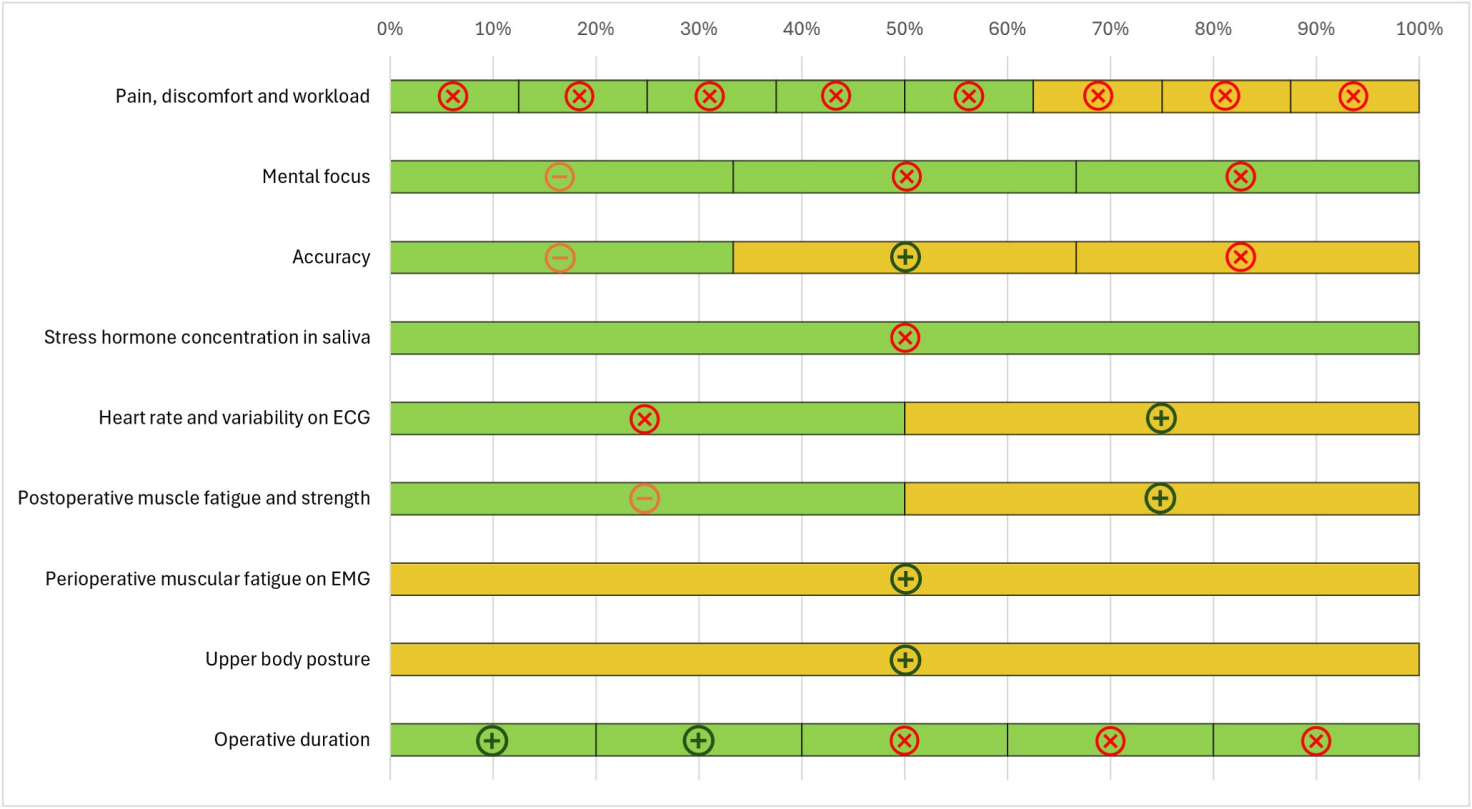
Summary of outcome results; each segment of a line represents one study; green segments represent a positive finding concerning an outcome with the intervention, yellow segments represent a neutral finding concerning an outcome with the intervention. Risk of bias is also represented for each outcome; a green “+” indicates a low risk of bias, an orange “–” indicates some concerns for bias and a red “X” indicates a high or serious risk of bias.

The literature search was conducted from inception until October 31, 2024, in the three following databases: MEDLINE, Embase and Google Scholar (no temporal or language restriction). A second search was performed in May 2026 to ensure that no additional relevant articles published since the initial search had been missed; however, no further eligible studies were identified. One additional article underwent full‐text assessment but was consequently excluded and is reported in the (Figure S1). Studies were collected, and duplicates were eliminated automatically and manually. Two authors (GH and FG) manually screened all studies by title and abstract in an unblinded manner. The excluded studies after full‐text assessment [18, 19, 20, 21, 22, 23, 24, 25, 26, 27, 28] are listed in the (Figure S1). The full texts were then retrieved and sorted a second time using the predefined selection criteria. The data from these studies were then extracted into a data collection form by two authors (GH and MH), which can be found in the (Figure S2). A systematic assessment of risk of bias was conducted by two authors (GH and MH), using the RoB 2 tool [29] for randomized studies and the ROBINS‐I (2016) tool [30] for non‐randomized studies. The corresponding assessments are presented in Figure 1 and in the (Figure S2). No formal assessment of publication bias could be performed due to heterogeneity of outcomes, the limited number of studies, and the absence of quantitative synthesis.

A descriptive synthesis of the included studies was conducted. For analyses addressing the objectives listed above, sufficiently comparable outcomes across studies (definition, measurement) were grouped and synthesized together. A summary of outcome categorization is provided in the (Figure S3), where outcomes are mapped according to their focus (patient‐vs. surgeon‐centered) and their nature (subjective vs. objective). A meta‐analysis was not conducted due to the heterogeneity of the studies and their small sample size.

### 2.1 Statistical Analysis

Descriptive statistics included absolute numbers and percentages, medians and range or interquartile range (IQR) when appropriate.

## 3 Results

### 3.1 Systematic Search

**FIGURE 2:**
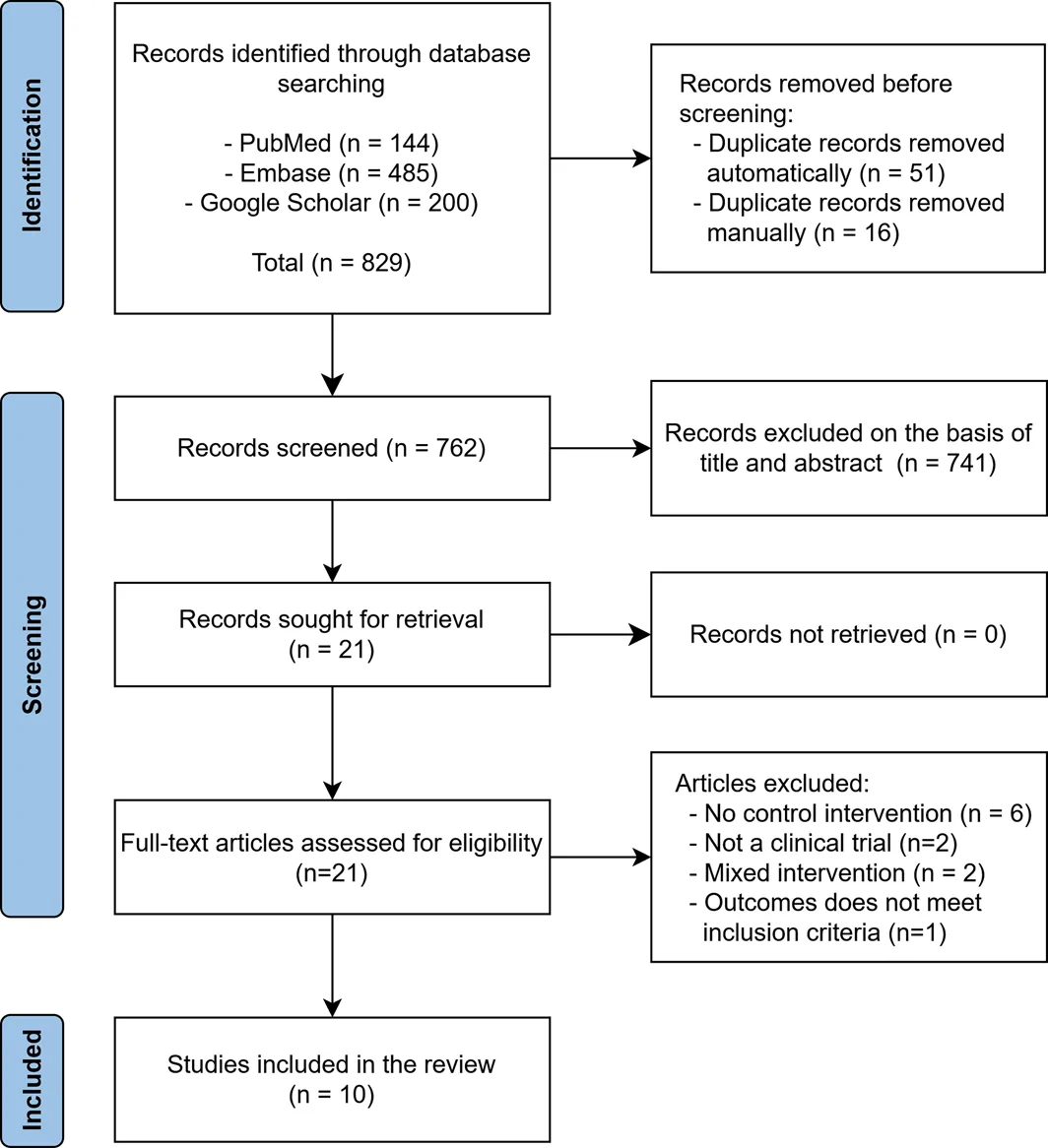
Flow diagram of the systematic search following the PRISMA template.

The flow diagram of the systematic search is summarized in Figure 2.

### 3.2 Study Characteristics

Details of the study characteristics can be found in the supplementary material.

Among the 10 included studies, 8 were crossover trials [31, 32, 33, 34, 35, 36, 37, 38] (randomized or non‐randomized) and 2 were randomized clinical trials [12, 39]. The study population included surgeons from various surgical subspecialties, including surgical trainees. The number of participants per study ranged from 2 to 73, with a median participant number of 19. The median number of operations with MBs per trial was 20 (range: 1–148), while 20 operations without MBs were performed in control groups (range: 1–193). Two trials were conducted in a simulated laparoscopy setting [37, 38] and eight in a real surgical routine [12, 31, 32, 33, 34, 35, 36, 39]. Risk of bias was rated as high or serious for 16 of the 26 assessed outcomes, as presenting some concerns for 3 outcomes, and as low for the remaining 7 outcomes, as depicted in Figure 1.

**FIGURE 3:**
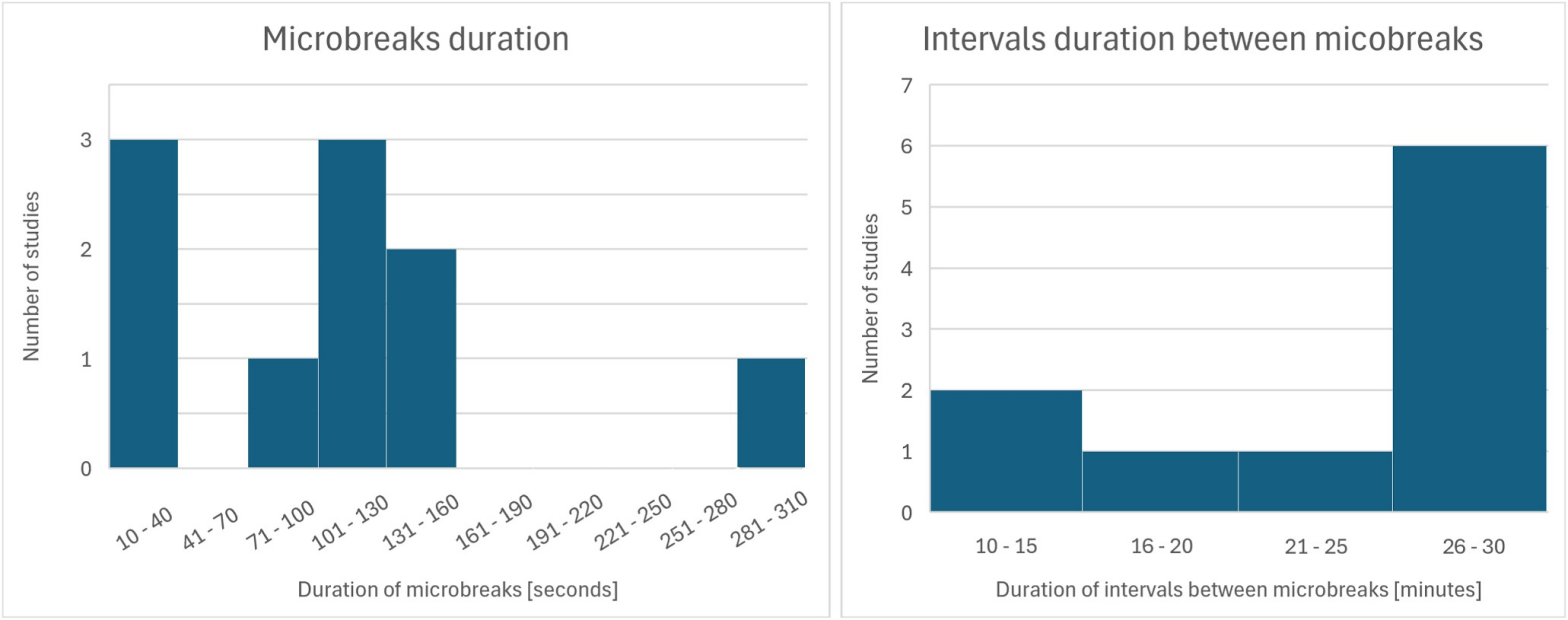
Repartition of the microbreaks duration and the intervals between them of the included studies.

There was significant heterogeneity between the included studies in terms of design, intervention, and outcomes. The interval duration between MBs, as well as the MB duration, varied considerably, as illustrated by the histogram in Figure 3. The median MB duration was 1.75 min (IQR 2) and the median interval duration between MBs was 25 min (IQR 10). In 7 studies, MBs consisted of active stretching of muscles and joints [31, 32, 33, 34, 35, 36, 39], in 1 study MBs were unstructured [12], and 2 studies evaluated active versus passive MBs in addition to a control group without MBs [37, 38]. Stretching exercises were guided by an audio script in two another studies [37, 38] and by a member of the study team in two studies [33, 35].

All studies using active stretching as in intervention (and providing details of the exercises) included stretching of the neck and shoulders [31, 33, 34, 35, 36, 37, 38, 39]. Most studies further incorporated more comprehensive exercises involving the lower limbs and trunk [33, 34, 35, 36, 37, 38].

The assessed outcomes were also largely heterogeneous, ranging from subjective (i.e., rating of perceived discomfort, perceived surgical performance, perceived workload (meaning the perceived level of strain or effort) [12, 31, 32, 33, 34, 35, 36, 38]) to highly objective outcomes (i.e., dosage of stress hormones, biometry with ECG [12]). These outcomes are detailed in the supplementary material.

### 3.3 Outcomes

The main results concerning the analyzed outcomes are synthetized in Figure 1, which details the proportion of positive findings (green) for each outcome. Further details can be found in the supplementary material.

Most studies (60%) revealed at least one positive outcome in favor of MBs. These were either subjective or objective and included perceived discomfort or performance measures.

Both trials conducted in a simulated laparoscopy setting failed to show any improvement of outcomes [37, 38].

No issues with adherence to MBs or stretching protocols were reported. However, two studies [31, 39] noted that surgeons tended to forget about the MBs during surgery and relied on the team to remind them. No undesirable effects on patients or surgeons have been reported in the MB groups. Interruption of the surgical workflow was not reported, besides one study [32] specifically assessing this aspect and revealing median scores of 2/10 for surgical flow disruption and 2.5/10 for distraction.

#### 3.3.1 Pain, Discomfort and Workload

80% of the studies included in this review assessed pain, discomfort or perceived workload, most of the time using visual analogic scales. Five out of these 8 studies showed an improvement in pain, discomfort or perceived workload after the MB intervention compared to the control groups [12, 31, 33, 34, 36]. The main anatomic regions relieved by the intervention were the neck, shoulders and arms.

The MBs duration did not seem to have a clear impact, since positive results were related to both long (5 min) [12] and short MBs (20 s) [31]. Similarly, reduction of pain has been shown with active stretching MBs but also in a trial with unstructured MBs [12].

Because this outcome is inherently subjective, the risk of bias was considered high in all studies.

#### 3.3.2 Mental Focus

All three studies that assessed mental focus showed some improvement with MBs [32, 33, 34]. One of the trials used a robust psychometric test evaluating concentration and performance pre‐ and postoperatively [12]. The intervention group gave a better performance (*p* < 0.05) leading to less errors (*p* = 0.06). The two other trials assessed mental focus subjectively by asking participants their opinion on the impact of MBs [32, 33], revealing positive results.

#### 3.3.3 Accuracy

One of the three studies evaluating manual dexterity right after surgeries showed an improvement with MBs [31], assessed via a task asking participants to cut along a star‐shaped track with scissors.

#### 3.3.4 Objective Outcomes

Several of the included studies used objective outcomes to evaluate the impact of MBs on surgeons, including reduced perioperative stress hormone concentrations (saliva cortisol and testosterone) [12] and reduced heart rate and variability with electrocardiogram monitoring [12], in contrast to Luger et al. [37] who did not find any difference of this assessment. There was also some discrepancy when assessing muscle fatigue and strength when asking surgeons to hold a 2.5 kg weight with their dominant arm fully extended as long as possible before and after surgery (with and without MBs): one study [31] showed a significant performance increase while the other revealed no improvement [35].

Finally, two more objective outcomes did not show any beneficial impact of MBs [37]: perioperative muscular fatigue measured by electromyogram (EMG) and upper body posture recorded with gravimetric inclination sensors.

Objective outcomes generally presented a lower risk of measurement bias due to their objective assessment methods. However, several studies [12, 31] were still evaluated as presenting “some concerns” or a high risk of bias for some outcomes due to limitations related to study design, selective reporting, or missing information regarding pre‐specified analysis plans.

#### 3.3.5 Operative Duration

All five studies that measured surgical duration found that MBs did not prolong the overall operative time, even when breaks lasted 5 min every 25 min [12, 32, 33, 34, 35]. Three of these studies [32, 33, 34] presented a “serious” risk of bias because surgical procedures were unmatched between groups, potentially introducing differences in procedural complexity and duration.

## 4 Discussion

This study suggests improved subjective and objective outcomes in surgeons taking ergonomic microbreaks. Microbreaks did not increase surgery time or disrupt the surgical workflow and come at no cost. Therefore, MBs can be considered in clinical routine, but the optimal modality has yet to be defined.

Ergonomists suggest targeted measures to reduce pain related to WMSDs, including adjustments both inside and outside of the operating room. A randomized controlled clinical trial assessing the effectiveness of a preventive programs proved that teaching of simple ergonomic principles to surgeons (i.e., correct monitor and table positioning, use of foot pedal, anti‐fatigue mats, footrests, and teaching of self‐treatment stretching exercises outside the OR) led to reduced lower back pain and analgesic consumption [40]. While assessment of pain, discomfort and perceived workload by validated questionnaires or visual analogic scales remains a valid method to evaluate the efficiency of MBs, more objective measures such as the consumption of analgesics may help to further elucidate their impact [40].

This systematic review revealed promising effects of MBs to decrease musculoskeletal pain, particularly in the neck, shoulders, and arms. These localizations are particularly at risk according to EMG studies objectivating muscular fatigue during surgery, with the middle trapezius muscles being the most heavily activated muscle group [41, 42]. The reduction of pain through MBs can be physiologically explained by several hypotheses, such as reduced tissue tension, therefore preventing stasis of the extracellular matrix [22] and vascular occlusion [43]. Furthermore, MBs allow for dynamic posture correction, offloading tissues and restoring anatomic loading of the articular surfaces [22]. Finally, MBs combined with active stretching promote articular lubrification and modulation of muscle sympathetic activity [22, 44]. The observed benefits on WMSDs are in line with ergonomic guidelines [14, 45], along with other modifiable factors that have been recommended to reduce musculoskeletal strain.

Operative duration is a critical parameter when considering incorporation of intraoperative breaks to respect the *Primum non nocere* principle. Based on this review, MBs may help surgeons to better anticipate next steps while improving physical (dexterity) and mental (focus) condition, thus enhancing overall and time‐effectiveness. Hence, MBs may be particularly useful for challenging, long and complex interventions. Indeed, Kromberg et al. [35] evaluated MBs during appendectomies and failed to show any benefits. Even though operative duration is not prolonged, it is understandable that taking a break may intuitively be perceived as a waste of time, thereby creating a barrier to further implementation of MBs. Other barriers may include skepticism to disrupt clinical routine, as well as the involuntary omission of MBs. Interestingly, some studies reported that surgeons easily forgot to take MBs during surgery unless reminded [39], or even enforced [31], by nursing staff. Therefore, implementing MBs with the involvement of the entire surgical team would likely improve adherence.

Additionally, MBs may provide opportunities for other purposes, such as teaching surgical trainees or debriefing the surgical situation with the team while stretching. Indeed, some studies [46, 47] revealed that structured, short intraoperative briefings improve patient outcomes and communication quality, thus benefiting patients as well as surgeons.

Interestingly, while improving post‐operative outcomes and pain in patients [48], minimally invasive surgical techniques inflict the opposite effect on surgeons, with open surgery causing significantly less pain compared to laparoscopy [2, 3, 6, 49, 50, 51, 52]. Laparoscopic surgery imposes ergonomic constraints, particularly due to the limited degree of freedom, long and rigid tools, and lack of direct visualization of the surgical field [53, 54]. Robotic surgery on the other hand appears to be more beneficial from an ergonomic standpoint [2, 3, 50]. Hence, intraoperative MBs may be particularly beneficial during laparoscopy, where physical strain is more pronounced. The discrepancy observed in the two negative studies using a simulated laparoscopy setup [37, 38] may hence be explained by an underestimation of the physical constraints experienced in real‐life procedures, leading to fewer observable benefits of MBs in artificial settings.

In addition to physical benefits, MBs appear to positively impact mental focus. However, this aspect was mainly subjectively assessed and should be evaluated through more objective tests to provide robust and reproducible results. In fact, several objective outcomes revealed promising findings. For instance, cognitive impact was assessed through stress biomarkers or salivary cortisol [12], which are highly relevant since excessive stress is known to impair surgeons' performance [55]. Similarly, improved muscle endurance and strength were reported [31], suggesting a functional benefit beyond subjective perception. There may also exist a beneficial impact on surgical accuracy, which is directly related to safe patient care and directly depends on mental focus, as improved concentration and reduced fatigue likely help maintain motor coordination [56]. A direct cause effect relationship on surgical precision however is matter of debate.

Together, these positive objective outcomes—ranging from physiological stress markers to functional performance—support the beneficial impact of MBs. However, risk of bias for some of these outcomes was considered high, and therefore the results should be interpreted with caution. Additionally, variations in MB definitions and protocols across studies (including duration, frequency, and timing) may have biased the reported outcomes and contributed to variability in the observed results.

Encouraging results were observed with different MB protocols, durations and intervals. Therefore, no single intervention can be recommended as gold standard. A meta‐analysis of 19 studies found that the longer the break, the higher the positive impact on performance [57]. Regarding the interval between breaks, this review suggests a trend toward more benefits with longer intervals, although difficult to assess given the small number of included studies. Comprehensive stretching exercises involving all relevant body parts appear most beneficial, but specific programs should also be proposed depending on the prevailing surgical approach and surgeon's symptoms.

### 4.1 Future Perspectives

This systematic review revealed several areas for future studies to explore, ideally yielding higher number of participants, longer follow‐up duration and optimized, standardized and comparable interventions. For the harmonization of future research, a standardized framework for the evaluation of MBs may be helpful, for example 2‐min MBs at 60‐min intervals, consisting of active stretching (consistent with the physiological mechanisms discussed above) of the lower limbs, trunk and especially neck and shoulders. Future studies should be realized in real surgical rather than simulated settings and involve the entire surgical team for best possible adherence. Objective outcomes such as hormonal stress markers, heart rate variability, muscle strength and accuracy should represent a particular study focus, while pain should also be assessed indirectly through analgesic consumption.

### 4.2 Limitations

This study has several limitations. First, due to the low number of studies, participants and surgeries, it is likely that some trials were underpowered to detect clinically significant differences. Negative studies may be the result of type II errors (low sample size) rather than ineffective interventions. Second, most studies were conducted over a single operative day or surgery impeding analysis of a longer‐term impact of MBs. Third, clinical outcomes have not been assessed and should be considered when designing future, adequately powered studies. Fourth, in the absence of quantitative pooling, the interpretation of findings relies primarily on the frequency of reported outcomes rather than on the magnitude or consistency of effects. Finally, the results of this systematic review are limited by an elevated risk of bias across several outcomes in the included studies.

## 5 Conclusion

In conclusion, this review highlights potential benefits of MBs for surgeons' wellbeing and performance. These benefits are observed through subjective perception but also objective measurements. MBs are safe, costless and do not increase operative duration.

Future studies should focus on more rigorous and standardized methodology, larger sample sizes, and long‐term follow‐up. Efforts should also focus on the “ideal ergonomic intervention” in terms of efficacy and applicability in clinical routine. Hereby the winner might not be a one‐fits‐all exercise but rather a set of exercises that can be chosen based on surgical approach and individual needs.

## Author Contributions

**Gabriel Heierli:** conceptualization, writing – original draft, methodology, visualization, formal analysis. **Fabian Grass:** conceptualization, writing – review and editing, methodology, visualization, supervision, formal analysis. **Luis C. Pereira:** writing – review and editing. **Jean Pellegrini:** validation. **David Fuks:** writing – review and editing. **Dieter Hahnloser:** validation. **Martin Hübner:** writing – review and editing, conceptualization, methodology, supervision, formal analysis.

## Funding

The authors have nothing to report.

## Conflicts of Interest

The authors declare no conflicts of interest.

## Supporting information


**Figure S1:** Excluded studies after full‐text assessment.

**Figure S2:** Data collection form with risk of bias assessment.

**Figure S3:** Summary of outcome categorization.

## Data Availability

The authors have nothing to report.
